# Application of Smoothing Methods for Determining of the Effecting Factors on the Survival Rate of Gastric Cancer Patients

**DOI:** 10.5812/ircmj.8649

**Published:** 2013-02-05

**Authors:** Hoda Noorkojuri, Ebrahim Hajizadeh, Ahmadreza Baghestani, Mohamadamin Pourhoseingholi

**Affiliations:** 1 Department of Biostatistics, Faculty of Medical Sciences, Tarbiat Modares University, Tehran, IR Iran; 2 Department of Biostatistics, Faculty of Paramedical Sciences, Shahid Beheshti University of Medical Sciences, Tehran, IR Iran; 3 Department of Biostatistics, Gastroenterology and Liver Diseases Research Center, Shahid Beheshti University of Medical Sciences, Tehran, IR Iran

**Keywords:** Proportional Hazards Models, Survival, Stomach Neoplasms

## Abstract

**Background:**

Smoothing methods are widely used to analyze epidemiologic data, particularly in the area of environmental health where non-linear relationships are not uncommon. This study focused on three different smoothing methods in Cox models: penalized splines, restricted cubic splines and fractional polynomials.

**Objectives:**

The aim of this study was to assess the effects of prognostic factors on survival of patients with gastric cancer using the smoothing methods in Cox model and Cox proportional hazards. Also, all models were compared to each other in order to find the best one.

**Materials and Methods:**

We retrospectively studied 216 patients with gastric cancer who were registered in one referral cancer registry center in Tehran, Iran. Age at diagnosis, sex, presence of metastasis, tumor size, histology type, lymph node metastasis, and pathologic stages were entered in to analysis using the Cox proportional hazards model and smoothing methods in Cox model. The SPSS version 18.0 and R version 2.14.1 were used for data analysis. These models compared with Akaike information criterion.

**Results:**

In this study, The 5 year survival rate was 30%. The Cox proportional hazards, penalized spline and fractional polynomial models let to similar results and Akaike information criterion showed a better performance for these three models comparing to the restricted cubic spline. Also, P-value and likelihood ratio test in restricted cubic spline was greater than other models. Note that the best model is indicated by the lowest Akaike information criterion.

**Conclusions:**

The use of smoothing methods helps us to eliminate non-linear effects but it is more appropriate to use Cox proportional hazards model in medical data because of its’ ease of interpretation and capability of modeling both continuous and discrete covariates. Also, Cox proportional hazards model and smoothing methods analysis identified that age at diagnosis and tumor size were independent prognostic factors for the survival of patients with gastric cancer (P < 0.05). According to these results the early detection of patients at younger age and in primary stages may be important to increase survival.

## 1. Background

In survival analysis, major interests are either to compare the failure time distribution function or to assess covariate effects on survival via appropriate hazards regression models. The Cox proportional hazards model is widely used in epidemiological research to examine the association between an exposure and a health outcome (1). In a typical approach to the analysis of epidemiologic data with a continuous exposure variable, the exposure is transformed to an ordinal or nominal polytomous variable and relative risk (RR) is modeled as a step function of the exposure. This approach is attractive because there are no constraints on the change in RR between exposure categories and because it is conceptually and computationally straightforward to implement. However, the selection of cut points used to define the exposure categories influences the shape of the dose-response relationship and this model sensitivity has raised concerns (2). Moreover, a step function does not take advantage of the information within categories (3, 4). Also, Cox proportional hazards model restricts the log hazard ratio to be linear in the covariates. A non-linear covariate effect may go undetected in this model. To avoid these pitfalls, as well as to avoid parametric constraints on the shape of the exposure-response curve, a variety of smoothing techniques have been recommended by epidemiologists (3, 5, 6).

Smoothing methods are widely used to analyze the epidemiologic data, particularly in the area of environmental health where non-linear relationships are not uncommon. Most of such applications fit cubic functions using splines (natural splines, restricted cubic splines, or penalized splines) or else, apply fractional polynomials (7). Several different smoothing techniques have been applied in environmental and occupational epidemiology. For example, smoothing splines have been used in generalized additive models to quantify the relationship of silica exposure and lung cancer (8) and to model air pollution and mortality (9, 10). Another common smoothing method, locally weighted regression smoother (LOESS), has also been used to model non-linear exposure-response relationships in generalized additive models relating air pollution and mortality (8, 11). Penalized splines (12) have recently appeared in several studies of occupational hazards and related health effects (11-17). Steenland and Deddens in 2004 described both penalized splines and restricted cubic splines in a review of alternative modeling approaches in occupational epidemiology (16). Restricted cubic splines (RCS) (18, 19) have also been applied in Cox models in both nutritional (20-22) and cancer epidemiology (16, 23, 24).

Gastric cancer (GC) is the second most common cancer in the gastrointestinal tract throughout the world (25). The patients are often diagnosed with advanced disease (26). Thus, diagnosis of the stomach cancer to a patient signifies the impending death. In fact, even among the medical professionals there is widespread belief that this diagnosis implies hopelessness. This attitude is a great deterrent to progress and is a sad one. Due to Japanese effort with scientific documentations, it is confirmed that the cancer of stomach is a curable disease (26). In the past two decades, because of the promotion of hygiene in Iran, death from different diseases has been reduced, but death rates due to cancers have remained as a major health problem among Iranian people (27). In Iran, the incidence is around 7300 cases per year, which is the most common cancer in men (28). During 2000-2005, incidence rate was highest in Northern provinces: Gilan, Mazandaran and Ardabil (29). Survival analysis is the modeling of time to event of death to evaluate the effects of treatment on survival time. It is important to determine the prognosis factors for patients with GC. Some potential clinicopathological factors such as age, tumor size, depth of invasion, distant metastasis, and pathologic type, have been evaluated to identify the factors affecting survival in these patients (30).

## 2. Objectives

The aims of the study were to assess the effects of prognostic factors on survival of the patients with GC using the smoothing techniques in Cox model and Cox proportional hazards. Also, all models were compared to each other in order to find the best one.

## 3. Materials and Methods

### 3.1. Study Population

This is a retrospective study of patients treated from February 2003 through January 2008, between 216 patients whom were admitted to the Taleghani hospital with a diagnosis of GC. The hospital is a referral center for gastrointestinal cancers, and all of the patients were diagnosed by endoscopy and biopsies. The exclusion criteria were the patients who had not completed document at hospital registry or treated out of the time February 2003 to January 2008 and the start point for survival time was the time of diagnosis which extracted from the patient’s document. The study protocol was approved by the ethics committee of the Research Center for Gastroenterology and Liver Disease of Shahid Beheshti Medical University. In the research center, all patients who register with gastrointestinal cancer are monthly followed for survival. The case of patient’s death was confirmed by contact with the patient’s family by telephone and clinical information was extracted from hospital documents. The Clinicopathological features were analyzed for GC patients were age at diagnosis, sex, pathologic distant metastasis, tumor size, histology type, regional lymph node metastasis and pathologic stage.

### 3.2. Statistical Analysis

In this study, Cox proportional hazards model and smoothing methods in Cox model were used for multivariate analysis. The smoothing methods considered in this paper are Penalized spline (P-spline), Fractional polynomial (FP) and restricted cubic spline (RCS). In this section, all models for analysis are reviewed.

### 3.3. Cox Proportional Hazards (Cox PH)

Currently, the most popular regression method for survival analysis in biomedical studies is the Cox proportional hazards model. The purpose of the model is to simultaneously explore the effects of several variables on survival. In this model, the effect of the covariates was to act multiplicatively on some unknown baseline hazard rate. Thus, under the Cox model, the hazard function for the failure time Ti associated with a p-vector of the covariates Zi=(zi1, …,zip) is defined as:

λ_(i ) (t)=λ_0 (t)exp⁡(β_1 z_i1+⋯+β_p z_ip)

Where is an unspecified baseline hazard function and is the regression coefficient, where k=1, 2, …, p. Estimation of proceeds through partial likelihood such that is not involved in the estimation of β_i. 1

### 3.4. Penalized Spline (P-spline)

The P-spline is a non-linear fit, but we can test how much of the effect is due to the linear part of the term versus the non-linear part. This is very similar to a post-hoc test for linear trend applied to a factor or class variable. The main point is that splines must be fit to a continuous predictor variable (31).

### 3.5. Fractional Polynomial (FP)

Fractional polynomial (FP) regression models are intermediate between polynomial and nonlinear models. The aim in using FP functions in regression is to keep the advantages of conventional polynomials, while eliminating (most of) the disadvantages. Put briefly, FP functions are similar to conventional polynomials in that they include powers of X, but non-integer and negative powers are also allowed. FP models usually give a better fit than conventional polynomials of the same degree, and even than those of higher degree. FP functions can be used with any generalized linear model and with Cox proportional hazards regression models for survival data. Examples are normal errors regression (multiple linear regression), (multiple) logistic regression and log-linear modeling of contingency table data which have ordered categories. In all of these a response variable Y is regressed on a single covariate X, or on several covariates X1,...,Xk.

We define the degree of an FP model as the number of terms in powers of X in the model and denote it m. Thus, for example y = b_0_+b_1_x-^1^ has degree m = 1 and y = b_0_+b_1_x^-1^+b_2_x^2^ has m = 2. We call the powers in the FP model p1, p2, etc., and denote the vector of powers as P. In the two examples just given we have p= -1 and P = (-1,2) respectively. It is uncommon to need models with m > 2, and so FP concentrates on models with m = 1 or m = 2 (32).

### 3.6. Restricted Cubic Spline (RCS)

Cubic splines are generally defined as piecewise-polynomial line segments whose function values and first and second derivatives agree at the boundaries where they join. The boundaries of these segments are called knots, and the fitted curve is continuous and smooth at the knot boundaries. To avoid instability of the fitted curve at the extremes of the covariate, a common strategy is to constrain the curve to be a straight line before the first knot or after the last knot (33).

In this study, at first, multivariate analysis of Cox PH model was fitted on all variables to determine the effective factors on survival of the patients with GC. Due to the suitability of spline models for continuous predictor variables, to compare the Cox PH model with P-spline, fractional polynomial and restricted cubic spline in Cox model from identified continuous effective variables in multivariate Cox PH model were used. These models were compared with each other by AIC (akaike information criterion) and LRT (Likelihood Ratio Test). Statistical analyses were performed using the computer program SPSS version 18.0 and R version 2.14.1. A P value of less than 0.05 was considered statistically significant.

## 4. Results

The mean age at diagnosis among the 216 patients was 50.23 ± 8.11 (rang: 26-69 years). The overall survival was 80% after one year, 40% after 3 and 30% after 5. Of the patients, 23 (10.6%) had pathologic distant metastasis, 175 (80.1%) had tumor size greater than 35mm, 136 (63%) diagnosed with advanced stage of GC, 164 (75/9%) with histology type of adeno carcinoma NOS and 26 (12%) in N3 level of regional lymph nodes metastasis. The results of the multivariate analysis of Cox PH model is also given in Table 1. Three covariates showed significant impact on the GC patients’ data in Cox PH models: age at diagnosis, tumor size and pathology stage.

**Table 1:** Multivariate Analysis of Prognostic Factors for GC Patients Usisng the Cox PH Model

Characteristics	RC^a^	SE	HR^a^ (95% CI)	P value
**Age at diagnosis**	0.052	0.015	2.113 (1.994-4.492)	0.042^c^
**Sex**				
Female^d^	-	-	1	-
Male	0.114	0.274	1.292 (0.522-1.526)	0.677
**Pathologic metastasis**				
Absent^d^	-	-	1	-
Present	0.480	0.342	1.453 (0.488-1.864)	0.889
**Tumor size**				
<35mm^d^	-	-	1	-
>35mm	0.548	0.277	1.730 (1.005-2.979)	0.048^c^
**Histology type**				
Other type^d^	-	-	1	-
Adenocarcinoma	-0.348	0.395	0.706 (0.349-1.427)	0.332
Signet cell carcinoma	-0.592	0.548	0.553 (0.189-1.619)	0.280
**Regional node metastasis**				
N1^b^ ^d^	-	-	1	-
N2^b^	0.289	0.418	1.335 (0.588-3.031)	0.490
N3^b^	0.662	0.543	1.939 (0.669-5.622)	0.223
**Pathologic stage**				
Early^d^	-	-	1	-
Adv	0.803	0.379	2.198 (1.070-4.513)	0.034^c^

^a^Abbreviations: RC, regression coefficient; HR, hazard ratio

^b^N1, Metastasis in 1-6 regional lymph nodes; N2, in 7-15; N3, &amp;gt;15 (according to SEER Summary Staging Manual 2000)

^c^Statistically significant at 0.05 level

^d^Reference group

**Figure 1:**
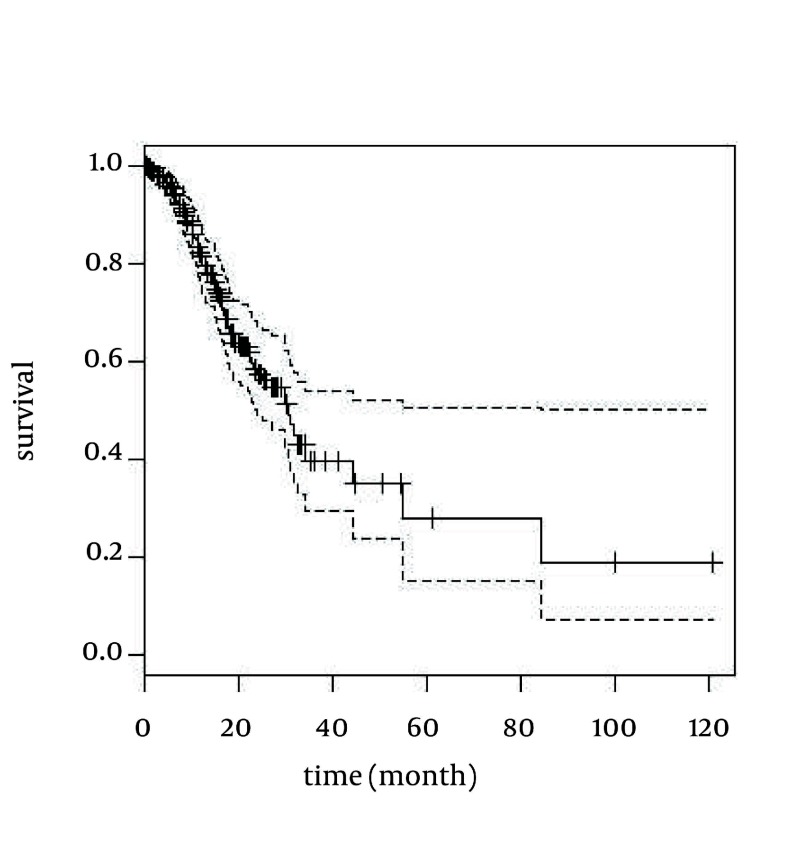
Survival Curve of GC Patients With 95% Confidence Interval for Smoothing Methods and Cox PH Model

Due to the suitability of spline models for continuous predictor variable, to compare the Cox PH model with P-spline models, fractional polynomial and restricted cubic spline in Cox model from indentified continuous effective variables in multivariate Cox PH model was used. The results of the comparing Cox PH and smoothing techniques in Cox models are also given in Table 2 and Figure 1.

**Table 2:** Results for Comparing Smoothing Methods and Cox PH Model in a Study of GC

Variable	Coefficient	SE	P value	LRT ^a^ (P value)	AIC ^a^
**Cox PH**				22.6 (0.00197)	689.4
Age at diagnosis	-0.0545	0.015	0.00028^c^		
Tumor size	-0.0262	0.013	0.04400^c^		
**P-spline**				22.6 (0.00197)	689.4
Age at diagnosis (linear)^b^	-0.0545	0.015	0.00028^c^		
Age at diagnosis (non)^b^			0.94000		
Tumor size (linear)	-0.0262	0.013	0.04400^c^		
Tumor size (non)			0.93000		
**Fractional polynomial**				22.6 (0.00197)	689.4
Age at diagnosis	-0.0545	0.015	0.00028^c^		
Tumor size	-0.0262	0.013	0.04400^c^		
**Restricted cubic spline**				22.9 (0.0112)	695.1
Age at diagnosis	-0.0567	0.0488	0.25000		
Tumor size	-0.0900	0.0450	0.04500^c^		

^a^Abbreviations: AIC, Akaike information criterion; LRT, Likelihood Ratio Test

^b^For the P-spline, the first term tests if the linear spline function is significant and the second term tests whether the non-linear component of the spline function is significant

^c^Statistically significant at 0.05 level

The analysis of Cox PH, P-spline and fractional polynomial in Cox model resulted in age at diagnosis and tumor size as prognostic factors on survival time of patients with GC independently (P < 0.05). The fact that non-linear part of the P-spline model has no significance shows that the non-linear effects of the model have been eliminated and the linear effects were good for these variables. Also, LRT, AIC and survival curve for these patients were equal in P-spline, fractional polynomial and Cox PH models but P-value, AIC and LRT in restricted cubic spline model was greater than other models. Note that the best model is indicated by the lowest AIC.

## 5. Discussion

Smoothing methods are widely used in epidemiologic study where non-linear relationship is not uncommon. We have compared smoothing methods (penalized spline, fractional polynomial and restricted cubic spline) in Cox model with Cox PH model and assessed the effects of prognostic factors on survival of the patients with GC. The Cox PH, P-spline and fractional polynomial models led to similar results, but P-value, AIC and LRT in restricted cubic spline was greater than other models. So, AIC showed a better performance for Cox PH, P-splines and fractional polynomial models comparing to the restricted cubic spline.

Numerous complex regression techniques are available to flexibly model the functional form of a continuous covariate’s effect on outcome. Particularly smoothing approaches that encompass a broad range of techniques and avoid assumptions of a particular functional form of a relationship between independent variables and outcome have been well-established in the statistical literature (34-36). Hollander and Schumacher in 2004 compared restricted cubic splines and fractional polynomials in Cox models through simulations and improved estimation of risk functions through bagging (37). In another report Govindarajulu et al. in 2007, applied penalized splines, restricted cubic splines, and fractional polynomials in survival models to data from two occupational cohort and compared results (5). In another study, Restricted cubic splines and penalized splines were found to be closer to each other than either was to the fractional polynomial in both datasets where they were used to model lung cancer mortality as a function of lifetime exposure (38) and to uranium, measured as radon progeny (39).

As we expected life expectancy significantly decreased with age at diagnosis. So, our finding was similar as previous reports (40-46). Also, a study performed in the United States showed that older age groups have a shortened life expectancy in comparison to young (46). In our results, sex had no effect on survival rate. Liu et al., Curtis et al. and Bako et al. indicated that there was no association between gender and survival of patients with early GC (47-49). Metastasis is another important prognostic factor of GC (50), however in our results no association was observed according to analysis. Size of tumor was another significant factor where affected the survival probability of patients in our analysis. This finding was in confirmed with those where pointed a higher hazard ratio of death for patients with larger tumor (47, 51, 52).

In the present study, the use of smoothing methods helps us to eliminate non-linear effects but it is more appropriate to use Cox proportional hazards model in medical data because of its’ ease of interpretation, capability of modeling both continuous and discrete covariates, accessibility of software packs, not being costly and time-consuming and no need of complicated programs and advanced computers. In this study, indicated that age at diagnosis and tumor size were associated factors for survival time in patients with GC. According to these results the early detection of patients at younger age and in primary stages may be important to increase survival.
